# Using blubber explants to investigate adipose function in grey seals: glycolytic, lipolytic and gene expression responses to glucose and hydrocortisone

**DOI:** 10.1038/s41598-017-06037-x

**Published:** 2017-08-10

**Authors:** Kimberley A. Bennett, Kelly J. Robinson, Simon E. W. Moss, Sebastian Millward, Ailsa J. Hall

**Affiliations:** 1 Division of Science, School of Science Engineering and Technology, Abertay University, Bell St, Dundee, DD1 1HG UK; 2 Marine Biology and Ecology Research Centre, Plymouth University, Drake Circus, Plymouth, PL4 8AA UK; 3 Sea Mammal Research Unit, Scottish Oceans Institute, University of St Andrews, St Andrews, KY16 8LB UK

## Abstract

Adipose tissue is fundamental to energy balance, which underpins fitness and survival. Knowledge of adipose regulation in animals that undergo rapid fat deposition and mobilisation aids understanding of their energetic responses to rapid environmental change. Tissue explants can be used to investigate adipose regulation in wildlife species with large fat reserves, when opportunities for organismal experimental work are limited. We investigated glucose removal, lactate, glycerol and NEFA accumulation in media, and metabolic gene expression in blubber explants from wild grey seals. Glycolysis was higher in explants incubated in 25 mM glucose (HG) for 24 h compared to controls (C: 5.5 mM glucose). Adipose-derived lactate likely contributes to high endogenous glucose production in seals. Lipolysis was not stimulated by HG or high hydrocortisone (HC: 500 nM hydrocortisone) and was lower in heavier animals. HC caused NEFA accumulation in media to decrease by ~30% relative to C in females, indicative of increased lipogenesis. Lipolysis was higher in males than females in C and HG conditions. Lower relative abundance of *11-β-hydroxysteroid dehydrogenase 1* mRNA in HG explants suggests glucose involvement in blubber cortisol sensitivity. Our findings can help predict energy balance responses to stress and nutritional state in seals, and highlight the use of explants to study fat tissue function in wildlife.

## Introduction

Knowledge of energy balance regulation is central to understanding how animals survive in changing environments. Appropriate regulation of energy balance is crucial for health, fitness and survival^1^ and depends on adipose tissue, which stores lipid and secretes hormones involved in regulation of appetite, metabolic rate, adipogenesis, fat deposition and mobilisation, reproduction, immunity and inflammation^2^. In wildlife, fat is required for insulation, buoyancy, streamlining and metabolic fuel^1, 3^. Predictable or erratic fluctuations in food availability and temperature, driven by natural or anthropogenic causes, cause adjustments to fattening strategy^4, 5^ and may alter adipose composition and function. Animals that naturally accumulate substantial fat reserves and forage at high trophic levels experience high lipophilic contaminant loads^6^, which can impact fat tissue function and energy balance^7^. Knowledge of adipose function and regulation is therefore vital to predict impacts of altered food availability and quality, contaminant exposure and stress on energy balance in wildlife species.

Manipulations via injection, implants, osmotic pumps, food or drink have been used in vertebrates to investigate behavioural, metabolic, hormonal, gene expression and fitness consequences of exposure to hormones involved in stress and energy balance^8–14^. Such studies may require captivity or lethal sampling, or reduce survivorship or reproductive output^15, 16^. Sample size^14^ and tissue volume are limited, the ability to perform simultaneous treatments is precluded, types and range of treatments that can be administered are restricted and underlying mechanisms cannot easily be identified^9, 17^. These issues are compounded for marine mammals, which have extensive fat stores, unique requirements for fat deposition and mobilisation, are often of conservation or management importance, but are difficult to access for whole animal experimental work and tissue sampling. Marine mammal cells have been used in short-term experiments^18, 19^ and subcutaneous fat from marine mammals can be obtained with minimal health impact^20^. However, although polar bear (*Ursus maritimus*) adipocytes can be maintained in culture^19^, elephant seal (*Mirounga angustirostris*) stromal vascular fraction cells do not accumulate fat or differentiate using standard protocols^21^. Field conditions and tissue volume also make adipocyte preparations challenging. Adipose explants, small pieces of *ex vivo* tissue in culture maintain structure, which can dictate tissue responses; better retain their original metabolic characteristics; are simpler to prepare and fewer cells are lost^22, 23^.

Understanding of energy balance regulation in phocid seals is largely based on associations between body mass changes and hormone levels, or small scale experiments and tracer studies^8–14, 24–26^ at the whole animal level. Grey seals (*Halichoerus grypus*) experience substantial fluctuations in fat reserves, from as low as 12% in juveniles and adults after moulting, to 33% in breeding adults, and 45% in weaned pups^27–30^. They rely heavily on fat as a metabolic fuel^29, 31^ and for insulation^32^. Males and females have different requirements and fattening patterns throughout the year^27, 30^. Body fat is vital for first year survival^33^, and adult reproductive fitness^34, 35^. Seals have unusual metabolic characteristics with some similarities to human diabetes^36^. Suckling grey seal pups do not suppress key enzymes involved in EGP and glucose levels are high when pups are both suckling and fasting^37, 38^. High glucose may support lipolysis^39^, and/or minimise fasting ketogenesis^25, 36^.

Glucocorticoids (GCs) are major regulators of energy balance in vertebrates, mediate responses to stress^17^ and have diverse effects on fat deposition, mobilisation, inflammation, differentiation and adipokine secretion^40^. GC effects on adiposity and lipolysis *in vivo* are confounded by counterregulatory hormone and neuronal inputs^40^. Tissue and cell level effects depend on the hormonal milieu and treatment duration^41^, glucocorticoid receptor (GR) abundance and 11β-hydroxysteroid dehydrogenase 1 (11βHSD1) activity, which locally converts inactive cortisone to active cortisol^42^. Cortisol increases during fasting in female and weanling northern elephant seals^43^ and has, therefore, been suggested to support lipolysis. However, circulating cortisol does not change in fasting grey seal pups, and adult male northern elephant seals^44, 45^, showing increased cortisol is not required to sustain fasting fatty acid supply. Treatment of fasting grey seal pups with a GC homologue increases protein not fat utilisation^8^, whereas treatment of elephant seals with adrenocorticotrophic hormone (ACTH) increases cortisol levels and is associated with elevated circulating non-esterified fatty acid (NEFA) levels in moulting, but not breeding individuals^12, 13^. The conflicting evidence to date concerning the role of glucose and GCs in fat regulation in phocids highlights the need for novel experimental approaches to explore underlying physiology in seals and other wild animals that have unusual fat regulation.

Here, we investigate the effect of glucose and hydrocortisone on glucose removal; lactate and NEFA accumulation; lipolysis, indicated by glycerol accumulation^46^; and relative mRNA abundance of key adipose genes in blubber explants from wild grey seals. We incubated explants from adult male and female seals overnight in control culture media (5.5 mM glucose) or elevated glucose (25 mM) or hydrocortisone (5.5 mM glucose + 500 nM hydrocortisone) conditions, and measured metabolite levels in the media and gene expression in the explants after 24 h exposure.

## Results

Body size and/or sex can influence metabolic properties of tissues. Here, male grey seals were significantly heavier (T test: T = 3.85, p = 0.007, df = 7.86), longer (T test: T = 5.10, p = 0.001, df = 5.90) and had greater girth (T test: T = 3.24, p = 0.01, df = 7.42) than females (Table 1). Condition index, a measure of body fatness, was not different between sexes (T test: T = 1.86, p = 0.12, df = 5.43).

**Table 1:** Body mass, length and girth, sex and time elapsed between sampling and onset of incubation in media for wild adult grey seals sampled at Abertay Sands in August 2013.

Sex	Animal	Mass (kg)	Length (cm)	Girth (cm)	Time from sample to incubation (min)
Females	1	165		153	206
	3	177.8	177	148	184
	4	170.4	181	145	166
	5	196.8	169.5	157	209
	6	190.6	170.4	152	201
	Mean (±S.D.)	180.2 ± 13.2	174.5 ± 5.5	151 ± 4.6	193 ± 18
Males	2	201	189	160	151
	7	192	189	156	169
	8	213.4	210	158	217
	9	218.6	200	171	224
	10	217.8	205	166	199
	Mean (±S.D.)	208.4 ± 11.5	198.6 ± 9.5	162.2 ± 6.2	192 ± 31
	Overall	194.3 ± 18.9	187.9 ± 14.8	156.6 ± 7.8	193 ± 24

### Glucose removal from media

**Figure 1:**
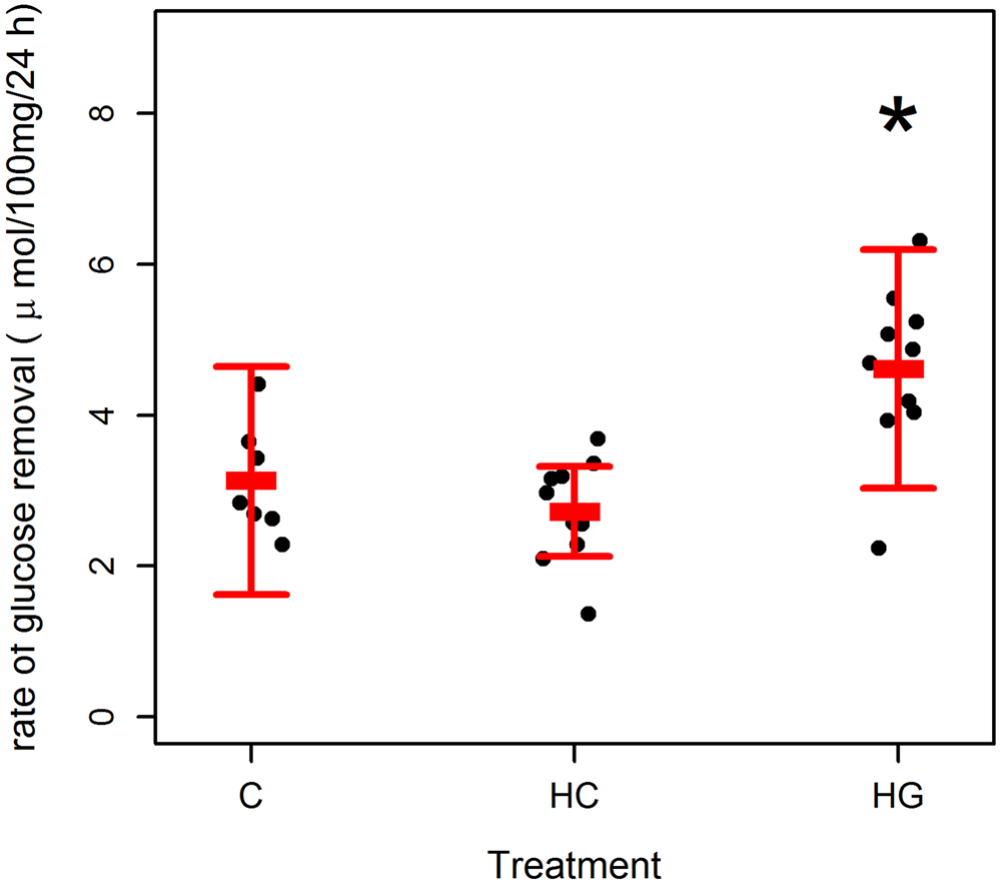
Effect of glucose and hydrocortisone on glucose removal from culture media by grey seal blubber explants. Glucose removal (Δ glucose 24 h^−1^ 100 mg^−1^) by 100 mg blubber tissue explants from grey seals (n = 10). Each point represents one animal. Bars represent mean (±s.d.). Tissues were minced and incubated in DMEM with 10% fetal bovine serum 5% penicillin and streptomycin, and glutamine. Explants from the same animal were supplemented with 5.5 mM glucose (C), 25 mM glucose (HG) or 5.5 mM glucose + 500 nM hydrocortisone (HC). Tissues and media were harvested after overnight (24 h) incubation. *Indicates where treatment was significantly different from C (LME: p < 0.001).

We quantified how much glucose was removed from the culture media by blubber explants over 24 h (Supplementary Table S1). Glucose removal differed significantly between treatments (LME: marginal R^2^_LME_ = 0.52; conditional R^2^_LME_ = 0.62; n (observations) = 27; n (animals) = 10). In this model, glucose removal was significantly higher (~2 fold) in HG explants than all other conditions (LME: T = 3.72; df = 15; p = 0.0021; Fig. 1), whereas glucose removal did not differ between C and HC explants (LME: T = 1.11; p = 0.286; df = 10). Sex (ANOVA: L ratio = 0.96; p = 0.328), body mass (ANOVA: L ratio = 0.02; p = 0.891) or condition (ANOVA: L ratio = 3.15; p = 0.07) did not affect glucose removal.

### Lactate accumulation in media

**Figure 2:**
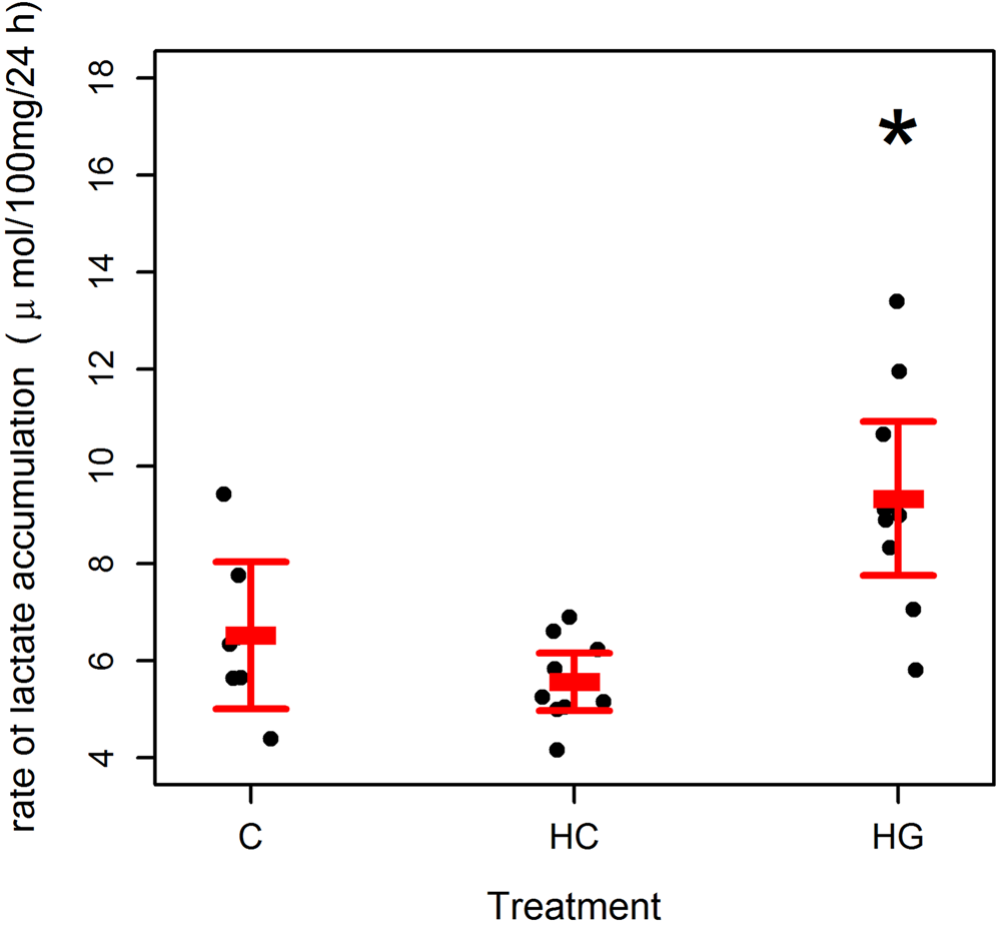
Effect of glucose and hydrocortisone on lactate accumulation in media by grey seal blubber explants. Lactate accumulation (Δ lactate 24 h ^−1^ 100 mg ^−1^) by 100 mg blubber tissue explants from grey seals (n = 10). Each point represents one animal. Bars represent mean (±s.d.). Tissues were minced and incubated in DMEM with 10% fetal bovine serum 5% penicillin and streptomycin, and glutamine. Explants from the same animal were supplemented with 5.5 mM glucose (C), 25 mM glucose (HG) or 5.5 mM glucose + 500 nM hydrocortisone (HC). Tissues and media were harvested after overnight (24 h) incubation. *Indicates where treatment was significantly different from C (LME: p < 0.001).

We investigated how glucose availability and GCs affect lactate production by seal blubber by measuring lactate accumulation in culture media. Log lactate accumulation was significantly higher (43–68%) in HG than in C explants (LME: T = 4.63; df = 15; p < 0.001; marginal R^2^_LME_ = 0.55; conditional R^2^_LME_ = 0.79; n (observations) = 27; n (animals) = 10; Fig. 2) or HC explants (LME: T = 7.61; p < 0.001; df = 15). In this model, log lactate accumulation tended to be lower in HC than C explants (LME: T = 2.07; p = 0.056; df = 15).

Inclusion of the rate of glucose removal to explain log lactate accumulation significantly improved the model fit (ANOVA: L ratio = 27.86; p < 0.001). There was a highly significant positive relationship between glucose removal and log lactate accumulation (LME: Log lactate accumulation = (0.084 * glucose removal) + 0.54; marginal R^2^_LME_ = 0.86; conditional R^2^_LME_ = 0.86; n (observations) = 27; n (animals) = 10; T = 7.06; df = 14; p < 0.001). When glucose utilisation was included as an explanatory variable in the model to explain lactate accumulation, the effect of treatment disappeared, showing that glucose removal accounts for the difference in lactate accumulation between conditions. Sex (ANOVA: L ratio = 2.11; p = 0.147), body mass (ANOVA: L ratio = 0.33; p = 0.567) and condition (ANOVA: L ratio = 2.20; p = 0.138) did not improve the model fit, showing that they did not affect lactate accumulation.

### Glycerol and NEFA accumulation in media

We used accumulation of glycerol, an index of lipolytic rate^46^, and NEFA in media to evaluate how blubber tissue fat mobilisation responds to glucose availability and GCs, and the modifying effects of sex, body mass or condition. Fat cells lack glycerokinase activity. Therefore, while NEFA can be re-esterifiedin culture, glycerol cannot^46^. NEFA accumulation in media thus reflects the dynamics between lipolysis and lipogenesis, whereas extracellular glycerol accumulation reflects lipolytic rate only.

**Figure 3:**
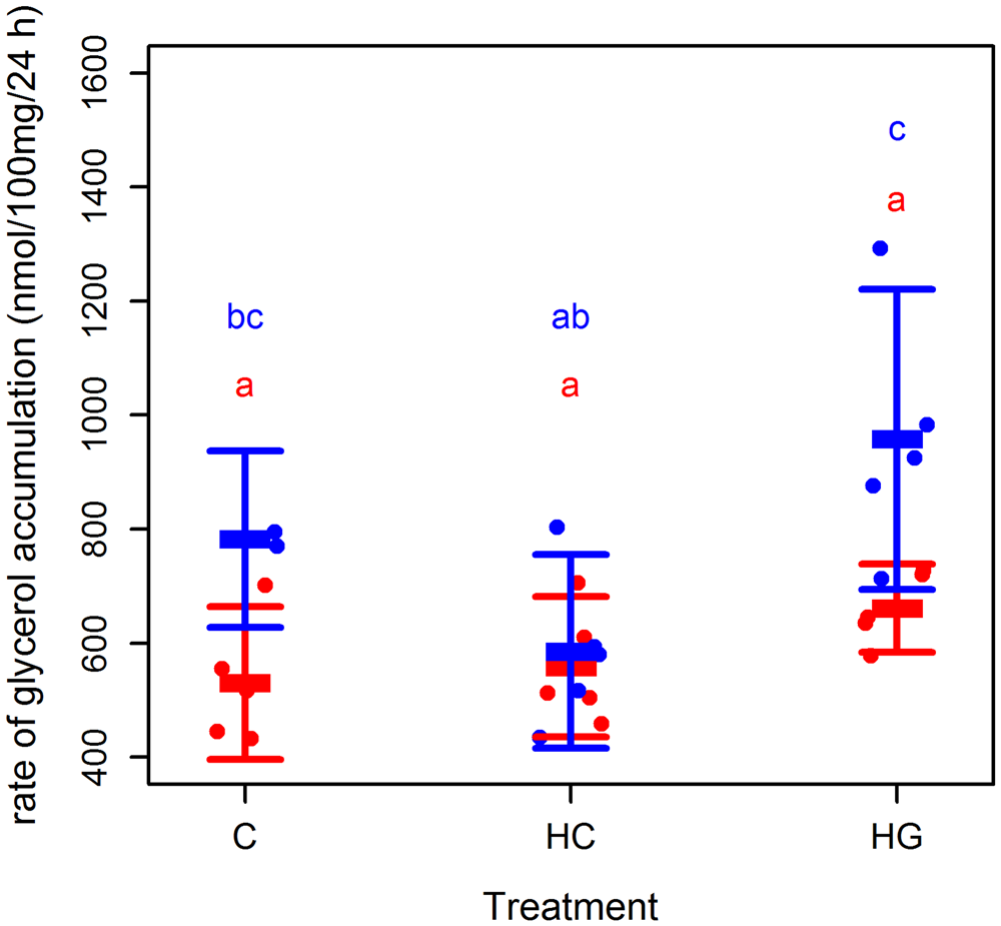
Effect of glucose and hydrocortisone on glycerol accumulation in media from grey seal blubber explants. Glycerol accumulation (Δ glycerol 24 h^−1^ 100 mg^−1^) by 100 mg blubber tissue explants from grey seals (n = 10). Each point represents one animal (red = female; blue = male). Bars represent mean (±s.d.). Tissues were minced and incubated in DMEM with 10% fetal bovine serum 5% penicillin and streptomycin, and glutamine. Explants from the same animal were supplemented with 5.5 mM glucose (C), 25 mM glucose (HG) or 5.5 mM glucose + 500 nM hydrocortisone (HC). Media was harvested after overnight (24 h) incubation. Different letters indicate where treatments were significantly different from each other (LME: p < 0.001).

The model that best described variation in log glycerol accumulation included an interaction between sex and treatment, and an additive effect of body mass (LME: marginal R^2^_LME_ = 0.74; conditional R^2^_LME_ = 0.74; n (observations) = 27; n (animals) = 10; Fig. 3). In this model, log glycerol production was higher in males than in females in C (LME: T = 3.50; p = 0.010; df = 7) and HG (LME: T = 4.07; p = 0.005; df = 7), but not in HC explants (LME: T = 1.70; p = 0.132; df = 7). In this model, log glycerol production in females did not differ between treatments (p > 0.05), whereas lipolytic rate was significantly higher in male HG explants compared with HC (LME: T = 4.70; p = 0.004; df = 7). There was no difference in males between C and HG (LME: T = 1.84, p = 0.089) or between C and HC explants (LME: T = 1.61; p = 0.131; df = 7). Body mass significantly improved the model fit (ANOVA: L ratio = 6.10; p = 0.014). There was a tendency for animals with higher body mass to have lower glycerol accumulation, irrespective of sex (LME: Log glycerol accumulation = (−0.003 * body mass) + 3.58; T = 2.28, p = 0.056). The mRNA abundance of *Atgl* (ANOVA: L ratio = 0.34; p = 0.557) or *Hsl* (ANOVA: L ratio = 0.40; p = 0.527), rate of glucose removal (ANOVA: L ratio = 2.53; p = 0.112) or condition (ANOVA: L ratio = 0.43; p = 0.514) did not explain any further variability in glycerol accumulation.

**Figure 4:**
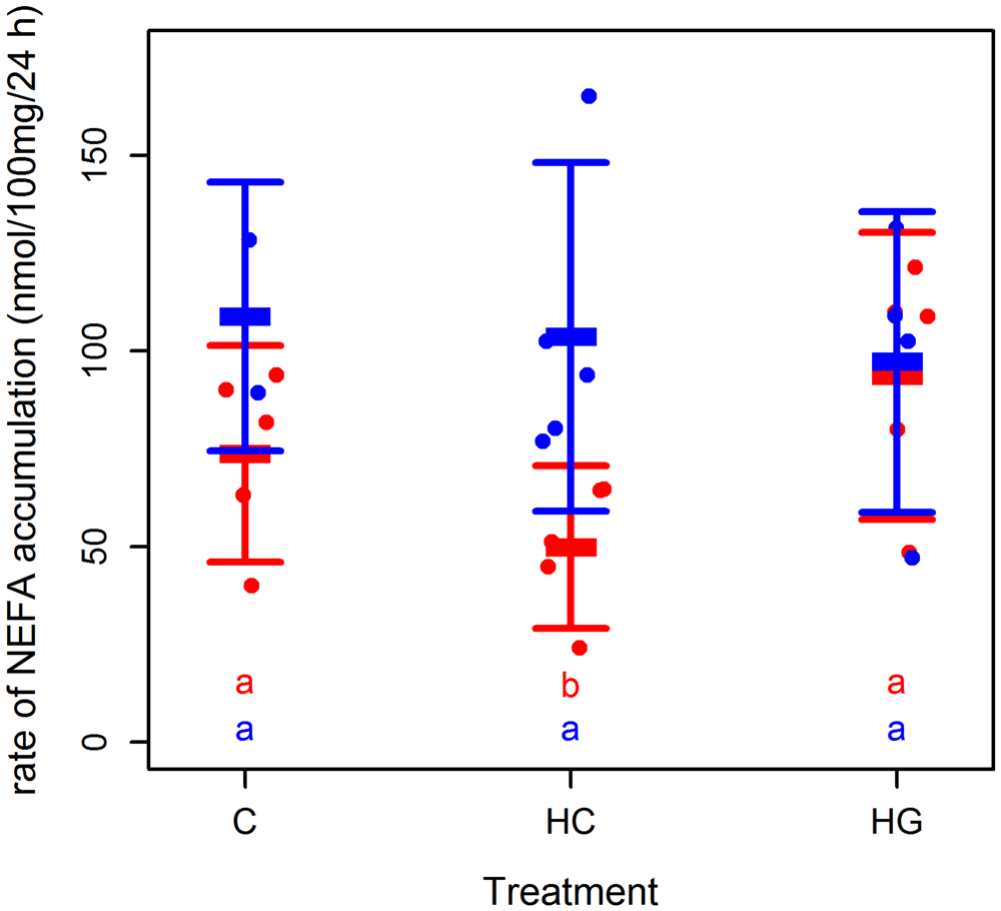
Effect of glucose and hydrocortisone on NEFA accumulation in media from grey seal blubber explants. NEFA accumulation (Δ NEFA 24 h^−1^ 100 mg^−1^) by 100 mg blubber tissue explants from grey seals (n = 10). Each point represents one animal (red = female; blue = male). Bars represent mean (±s.d.). Tissues were minced and incubated in DMEM with 10% fetal bovine serum 5% penicillin and streptomycin, and glutamine. Explants from the same animal were supplemented with 5.5 mM glucose (C), 25 mM glucose (HG) or 5.5 mM glucose + 500 nM hydrocortisone (HC). Media was harvested after overnight (24 h) incubation. Different letters indicate where treatments were significantly different from each other (LME: p < 0.05).

An interaction between sex and treatment best described the accumulation of NEFA in the culture media (LME: marginal R^2^_LME_ = 0.38; conditional R^2^_LME_ = 0.82; n (observations) = 27; n (animals) = 10; Fig. 4). In this model, explants from males showed no difference in NEFA accumulation in media between treatments (LME: C v HG: T = 0.73; p = 0.479; df = 13; C v HC: T = 0.28; p = 0.785; df = 13; HG v HC: T = 0.65; p = 0.530). In contrast, explants from females showed a tendency for NEFA accumulation to increase by ~30% in HG conditions relative to C (LME: C v HG: T = 2.01; p = 0.066; df = 13) and to decrease ~30% relative to C under HC conditions (LME: C v HC: T = 2.40; p = 0.032; df = 13). NEFA accumulation was therefore significantly higher in HG than HC explants from females (LME: C v HC: T = 4.41; p < 0.001; df = 13). NEFA accumulation did not differ between male and female explants in C (LME: T = 1.62; p = 0.146; df = 8) or in HG conditions (LME: T = 0.19; p = 0.851; df = 8). Females had significantly lower NEFA accumulation in media than males in HC conditions (LME: T = 2.92; p = 0.019; df = 8). Including body mass (ANOVA: L ratio = 1.65; p = 0.198), condition (ANOVA: L ratio = 3.07; p = 0.198), relative mRNA abundance of *Atgl* (ANOVA: L ratio < 0.01; p = 0.991) or *Hsl* (ANOVA: L ratio = 0.37; p = 0.543) or glucose removal rate (ANOVA: L ratio = 0.01; p = 0.905) did not explain any more of the variability in NEFA accumulation in media.

### Correlation between rates of accumulation and removal

We investigated whether rates of glucose removal and accumulation of lactate, NEFA and glycerol were correlated within each treatment (Table 2). Glucose removal and lactate accumulation were correlated in each condition. Lactate and glycerol accumulation were positively correlated in HG and HC treatments, but not in controls. There were no other correlations between rates of metabolite removal or accumulation.

**Table 2:** Correlation between glucose (Glu) removal and lactate (lac), glycerol (gly) and NEFA accumulation in media from grey seal blubber explants (n = 10) incubated for 24 h in 5.5 mM glucose (C), 25 mM glucose (HG) or 5.5 mM glucose + 500 nM hydrocortisone (HC). Significant (p < 0.05) correlations are indicated in bold font.

	C			HG			HG		
	R	T	p	R	T	p	R	T	p
**Glu x lac**	**0.962**	**7.97**	**0.0005**	**0.852**	**4.61**	**0.002**	**0.752**	**3.22**	**0.0012**
Glu x gly	0.495	1.27	0.259	0.515	1.10	0.128	0.510	1.68	0.132
Glu x NEFA	0.647	1.90	0.116	−0.227	0.66	0.528	−0.407	1.26	0.244
Lac x gly	0.389	0.944	0.388	**0.788**	**3.63**	**0.007**	**0.678**	**2.61**	**0.031**
Lac x NEFA	0.598	1.67	0.156	−0.22	0.64	0.543	−0.032	0.09	0.931
Gly x NEFA	0.132	0.299	0.777	−0.457	1.45	0.184	−0.05	0.14	0.891

### mRNA abundance

**Figure 5:**
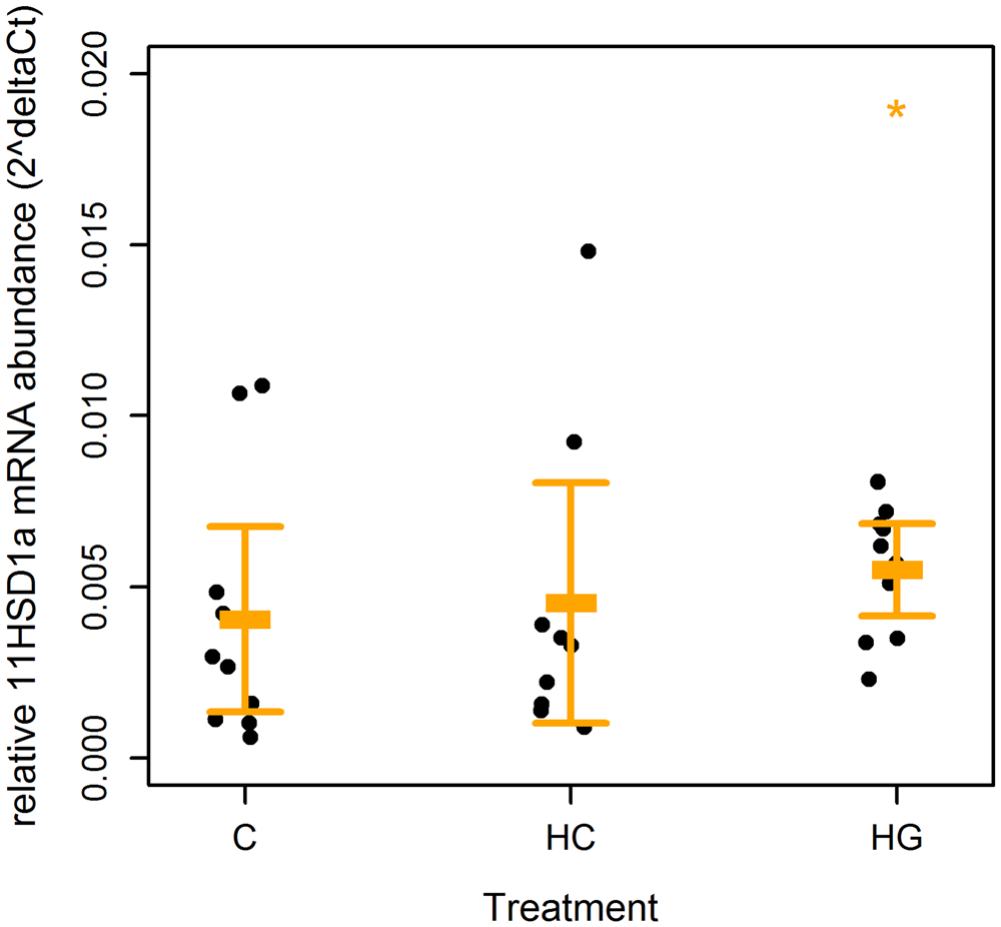
Effect of glucose and hydrocortisone on relative *11βhsd1* mRNA abundance of grey seal blubber explants. Relative *11β hydroxysteroid dehydrogenase 1 (11βhsd1)* mRNA abundance (2^ΔCT^) by 100 mg blubber tissue explants from grey seals (n = 10). Each point represents one animal. Bars represent mean (±s.d.). Tissues were minced and incubated in DMEM with 10% fetal bovine serum 5% penicillin and streptomycin, and glutamine. Explants from the same animal were supplemented with 5.5 mM glucose (C), 25 mM glucose (HG) or 5.5 mM glucose + 500 nM hydrocortisone (HC). Tissues were harvested after overnight (24 h) incubation. *Indicates where treatment was significantly different from C (LME: p < 0.001).

We quantified the relative transcript abundance^47–49^ of metabolic genes in blubber explants to evaluate the effects of glucose availability and GCs and the modifying effects of sex, body mass or condition, on adipose tissue function. Relative abundance of *11βhsd1* mRNA was significantly lower in HG than C for some individuals (LME: R^2^_LME_ marginal = 0.13; R^2^_LME_ conditional = 0.59; n (observations) = 29; n (animals) = 10; df = 17; T = 2.65; p = 0.017; Fig. 5), and tended to be lower than in HC (T = 2.07; p = 0.054). Relative abundance of *11βhsd1* mRNA was not different between HC and C explants (T = 0.49; p = 0.628). There were no changes in relative mRNA abundance of *GR, PPARγ, Atgl* or *Hsl*, *adiponectin* or *leptin* genes in response to any treatment (Table 3). Relative *Atgl* abundance tended to be negatively related to body mass (LME: Log *Atgl* = −0.006 mass + 1.07; T = 2.20; p = 0.056; df = 8; R^2^_LME_ marginal = 0.15; R^2^_LME_ conditional = 0.15; n (observations) = 30; n (animals) = 10), but the relationship was weak. There were no other differences between sexes or effects of body mass or condition on relative expression of target genes (LME: p > 0.05).

**Table 3:** Mean (±S.D) fold difference in relative mRNA abundance of each gene of interest (GOI) relative to the control (5.5 mM glucose) condition. Blubber explants derived from biopsy cores from adult grey seals (n = 10) were divided into 100 mg portions of tissue and exposed to 5.5 mM glucose (control); high glucose (HG: 25 mM) or high hydrocortisone (500 nM + 5 mM glucose: HC) overnight and harvested by snap freezing the following day. GOI Ct values were divided by the geometric mean of two non-regulated reference genes (*CycA* and *S9*) and log transformed. The effect of treatment on each GOI was investigated in an LME, with individual as a random effect. T, p, and marginal (mar) and conditional (cond) R^2^_LME_; n (individuals); n (observations) and degrees of freedom (df) are given for each gene of interest. Where n (individuals) is less than 10 there was insufficient RNA to run all GOI. *Atgl* = *adipose triglyceride lipase*; *Hsl* = *hormone sensitive lipase*; *Pparγ* = *peroxisome proliferator activated receptor γ*; *Gr* = *glucocorticoid receptor*.

GOI	HG			HC			R^2^_LME (mar)_	R^2^_LME (cond)_	n (ind)	n (obs)	df
	fold	T	p	fold	T	p					
*Atgl*	−0.85 ± 1.78	1.62	0.121	−0.20 ± 1.63	0.23	0.819	0.06	0.09	10	30	18
*Hsl*	−0.21 ± 1.07	0.37	0.718	−0.22 ± 1.06	0.79	0.440	0.02	0.02			
*Pparγ*	0.04 ± 1.14	0.61	0.549	−0.36 ± 1.07	0.82	0.425	0.03	0.03			
*Gr*	0.40 ± 0.98	0.13	0.901	0.34 ± 1.02	0.51	0.615	0.02	0.06	10	29	17
*Adiponectin*	−11.64 ± 32.58	1.11	0.284	−3.32 ± 11.78	0.47	0.649	0.10	0.23	8	24	14
*Leptin*	0.26 ± 1.11	0.22	0.825	0.22 ± 1.15	0.56	0.584	0.03	0.03			

## Discussion

Glucose removal and lactate accumulation in media were highly correlated and stimulated by increased glucose availability, as expected from human and rodent studies^50, 51^. This supports *in vivo* elephant seal studies showing responses of circulating glucose and lactate to exogenous ACTH are inversely related and tightly coupled^13^. Since HG increased glucose removal and lactate accumulation here, it likely also increased TAG synthesis because lipogenesis parallels increased lactate production. All products of glucose metabolism increase 10 fold in the ‘fed’ state^52^, which may also explain the correlation between lactate and glycerol accumulation under HG and HC conditions.

Blubber explants produced substantial amounts of lactate in basal conditions. Increased lactate accumulation under high glucose conditions may have resulted from local hypoxia caused by limited access of oxygen to central regions of the explants and/or glucose-stimulated glycolysis or acetyl-coA generation. Subcutaneous fat is a significant source of whole body lactate production in humans and rodents, contributing 10–30% of glucose metabolism^53^. In isolated adipocytes, 50–70% of glucose uptake is converted into lactate by lactate dehydrogenase (LDH)^51, 54^. Hypoxia and lactate production could be normal for seal blubber because adipocyte sizes often exceed oxygen diffusion distances, fat is poorly vascularised and has lower oxygen levels than other tissues^55^. However, oxygen is very soluble in fat, such that blubber may not have a limited oxygen supply either in the experimental conditions here or *in vivo*. Interestingly, when there is an abundance of acetyl co-A, such as during increased glucose influx, pyruvate dehydrogenase is inhibited and lactate production is increased as a result of increased flux through LDH, even when metabolism is aerobic^56^.

LDH activity in rodent adipose tissue is reduced by fasting, diabetes and hypophysectomy^57^. Plasma LDH activity in fasting elephant seals is positively related to insulin levels and seals can modulate their insulin sensitivity based on their fed or fasting state^10, 11, 36, 58^. Insulin sensitivity may be greater in recently fed animals than during an extended fast. We sampled adults that were hauled out in August, when grey seals are in a fattening state^27, 30^ and we have no information on how recently they had fed. Grey seals frequently return to land to haul out for one or more days between foraging trips, but may forage while close to haul out^59^. The animals in this study could have been fasting for several days and were certainly postprandial when caught and sampled. However, they were not undertaking an extended fast typical of the breeding or moulting period. In addition, the explants may have been exposed to low levels of insulin in foetal calf serum. Prior and ongoing insulin exposure of the explants may thus have facilitated glucose uptake through GLUT4 and lactate production through LDH stimulation.

High whole-body lactate production in fasting elephant seals has been suggested to maintain elevated EGP and avoid excessive ketosis^25^. The origin of the lactate has not been identified. High EGP in fasting elephant seals does not derive from glycogen^58^ or protein catabolism^60^. Muscle can produce substantial amounts of lactate during exercise^61^, but does not account for a large proportion of lactate production at rest, even when glucose is elevated^62^. Our data suggest that adipose-derived lactate may contribute significantly to whole body lactate production in seals, which could be substantial given their high fat diet, relative insulin insensitivity^10, 11, 36^ and considerable adipose reserves^29–31^. We suggest high EGP in seals could protect against lactic acidosis that may otherwise arise from high rates of blubber glycolysis.

In rodent and human studies adipose lactate production falls as adipocytes shrink, because smaller fat cells experience reduced hypoxia and/or higher mitochondrial density and have a greater capacity for oxidative phosphorylation^51, 53^. In fasting weaned elephant seals, mass specific lactate production and contribution to gluconeogenesis remain constant^25^. The fall in absolute lactate production concomitant with body mass reduction *in vivo* could be a result of reduced fat cell size and/or a fall in insulin and insulin sensitivity.

HC caused a reduction in lactate accumulation here, which is consistent with decreased circulating lactate after ACTH treatment in fasting elephant seals^12, 13^ and suggests that blubber may contribute to whole animal ACTH responses. GCs inhibit adipocyte glucose uptake and lactate production in rat and human tissues *in vitro* ^63, 64^. No change in glucose removal was seen here in response to 24 h HC exposure, but the sampling rate in this experiment precluded detection of small or transient changes in glucose removal.

Glucose stimulation of lipolysis occurs over 1–2 h in rat tissue^65^. The highest lipolytic rates occur in the first 10 minutes and require insulin, glucose and noradrenaline^39^. This short time frame may explain lack of correlation between glucose removal and glycerol accumulation over 24 h here. In response to high glucose we saw a tendency for a higher rate of NEFA accumulation in females but not males, and neither sex showed an increase in glycerol accumulation. NEFA and glycerol accumulation were not correlated. Glycerol is an index of lipolytic rate in isolated fat tissue and culture because it cannot be re-esterified by adipocytes, whereas NEFA can^46^. In the absence of glycerol accumulation changes, the higher NEFA accumulation in females when glucose is elevated is likely a result of lower lipogenesis, because glucose can inhibit cytosolic phosphoenolpyruvate carboxykinase (PEPCK-C) activity and expression. PEPCK-C catalyses glycerol-3-phosphate synthesis from glycerol and allows re-esterification of NEFA despite net glycerol efflux^66^.

In fasting elephant seal pups, the NEFA response to a glucose tolerance test depends on when the test is administered during fasting^11^. The net lipolytic response of fat tissue to elevated glucose is therefore context dependent, and this is also true of the response to GCs. Indeed, recent nutritional state, age and fat cell size influence tissue responses *in vitro* ^66^. The lack of increased NEFA or glycerol accumulation in HC treated explants supports *in vivo* experimental work in grey seal pups showing GCs alone do not stimulate fat catabolism^8^. The 500 nM hydrocortisone dose used here, which reflects circulating cortisol during acute stress in this species^44^, had sex specific effects on indices of fat metabolism. Glycerol accumulation in media from male HC explants was lower than those in HG conditions, whereas glycerol accumulation from female explants did not change. However, HC drove a decrease in NEFA accumulation in media from female but not male explants. These data suggest males modulate lipolytic activity, whereas females increase lipogenesis without changing lipolysis in acute response to HC. However, GCs tend to decrease *PEPCK-C* gene expression and activity in adipose cells to reduce glyceroneogenesis, which should reduce lipogenesis^66^. Acute effects of GCs tend to be lipogenic whereas longer lasting effects tend to be lipolytic^41^. Acute ACTH challenge induces expression of genes involved in both adipogenic and lipolytic pathways in fasting elephant seal pups^14^. ACTH challenge results in no change in NEFA in elephant seals during breeding and 20–60% increased NEFA during moulting^12, 13^. GCs can potentiate the lipogenic effects of insulin^41^, which may have been present here in the culture media, and dictates whether HC produces a lipolytic or lipogenic response in fat. Blubber from fasting, juvenile or moulting animals may thus respond to HC treatment differently to the recently fed and fattening adults sampled here, on the basis of their insulin sensitivity and prior insulin exposure. Investigation of tissue and whole animal metabolic responses, both within and between species, during the breeding and postweaning fast, rapid fat accretion during suckling, in moulting and juvenile animals and in response to a wider range of hormone and metabolite conditions will provide informative comparisons and help identify functional consequences of measured hormone levels in wild seals.

Males had a higher basal lipolytic rate than females, which corresponds with findings from humans^67^. Male juvenile elephant seals have higher rates of energy expenditure and spare protein more effectively than females^68^. Higher lipolytic rates in adult male grey seals likely contribute to sex differences in timing and extent of fat accumulation throughout the year^27, 30^. Heavier animals had lower lipolytic rates and relative *Atgl* mRNA abundance. This is consistent with the finding that ATGL is the major lipase in elephant seals^69^ and emphasises its importance in fat regulation and energy balance in seals.

Glucose appeared to downregulate relative mRNA abundance of *11βhsd1* in some individuals, although the variability explained by glucose was low. Hypoxia inducible factor 1 α (HIF1α) lowers 11βHSD1 expression in adipose tissue and adipocytes^70^ and could have been induced here by localised hypoxia or ‘pseudohypoxia’ caused by high glucose influx, which stabilises HIF1α^52^. If elevated glucose can minimise GC effects on fat tissue *in vivo* in some individuals, it may modulate tissue responses during stress. The relative mRNA abundance of other adipose-specific genes investigated did not change with treatment, sex or body condition, an index of fatness. We did not measure relative mRNA abundance of lipoprotein lipase (LPL: a major determinant of tissue fatty acid uptake in seal blubber^71^), diacylglycerol transferase (DGAT: important in intracellular re-esterification of diacylglycerol in adipose^72^), fatty acid synthase (FAS: regulates de novo lipogenesis from acetyl -co-A^72^), or PEPCK-C, which may help to identify lipogenic responses. Changes in transcription or activity in these and other lipogenic genes occur in response to GCs in humans^66^ and ACTH in elephant seals^14^, and may contribute to observed sex differences in net fatty acid accumulation in media and response to HC here. Glucose and GC treatment induce changes in abundance of the genes of interest measured here within 4–24 h in isolated adipocytes and exposed tissue from humans and rodents eg.^73, 74^ and in ACTH challenged elephant seal pups^14^. These changes did not occur here, perhaps because explants contain mostly mature adipocytes and few differentiating cells and/or did not have input from other hormones. Explant heterogeneity likely contributed to gene expression variability and poor signal to noise ratio. However, our data shows how an *in vitro* approach may help to tease apart the mechanisms underlying whole animal responses to experimental manipulations when hormone or metabolite clamping may be challenging to accomplish.

Short term explant culture is clearly a viable method to complement whole animal work in understanding molecular and cellular physiology of fat tissue in seals. Further work is required to assess heterogeneity of tissue composition and structure within and between explants, to refine culture methods to better reflect physiological conditions, and to extend the duration of explant viability. Explants reduce the number of animals sampled, permit complex exposures that would not be possible *in vivo* to tease apart mechanisms, and increase statistical power by pairing samples. Our approach will allow comparison of human, rodent and seal tissues under identical conditions to determine the extent to which seals model responses in diabetic and obese patients. Use of explants will also be of value in other wildlife species for which adipose culture has not yet been developed, but from which fat samples can be returned to the laboratory quickly and maintained in appropriate tissue culture facilities.

## Methods

### Animal capture and sample collection

Wild, adult grey seals were captured using a standard seine net technique at Abertay sands, Fife, UK in August 2013. Five males and five females were restrained in hoop nets prior to anaesthesia using a mass specific intravenous dose of zoletil^TM^. Morphometric measures (girth, length and body mass) were obtained and condition (mass/length^2^) was calculated. Full depth, 6 mm blubber biopsies were taken^26^, followed by 1 ml 10 kg^−1^ intramuscular terramycin^TM^ (Pfizer Ltd) injection. Animals recovered from anaesthesia fully before release. All capture and handling procedures and experimental protocols were approved by the Animal Welfare and Ethics Committee at the University of St Andrews, and performed under Home Office project licence 60/4009 and the annual licence issued to the Sea Mammal Research Unit in accordance with the UK Marine (Scotland) Act 2010, and the UK Animals (Scientific Procedures) Act 1986.

### Blubber explant preparation and exposure

Biopsies (~400 mg and ~3–4 cm length) were transferred to warm sterile Krebs-Ringer solution (KHPO_4_; 0.55 g/l; 120 mM NaCl; 0.25 g/l MgSO_4_.7H_2_O; 0.84 g/l NaHCO_3;_ pH 7.4; 0.11 g/l CaCl_2_; 30 mM HEPES; 1% BSA; 200 nM adenosine (all chemicals supplied by Sigma- Aldrich)) in 15 ml Falcon tubes and transported within 2–4 hours to the laboratory. Explant preparation was adapted from established methods^22, 23^. Blubber was washed twice in fresh Krebs-Ringer. Adhering skin, muscle or connective tissue was removed. Biopsies were blotted, weighed and minced into pieces of 5–10 mg. Portions of ~100 mg were weighed out into 2 ml sterile DNAase and RNAse free tubes. Immediately, 1 ml of pre-warmed media (see supplementary material), containing 5.5 mM glucose (control: C); 25 mM glucose (HG); or 5.5 mM glucose with 500 nM hydrocortisone (HC) was added to the appropriate tubes, which were incubated overnight at 37 °C at 5% CO_2_. Each aliquot contained a mixture of explant pieces from different biopsy depths to prevent potential bias caused by blubber stratification. Duplicate exposures were performed for each animal. Media was drawn off after 24 h and stored at −20 °C. Explants were snap-frozen and stored at −80 °C.

### Biochemical analysis

We measured glucose, lactate, glycerol and NEFA in media using Randox (County Antrim, UK) kits and standards in an RX Monza (Randox) Clinical Chemistry analyser (Model number: 328-14-0914). QC measurements lay within <  +/− 15%. Intra and inter assay variability for samples was < 10%. Rates of glucose removal and accumulation of lactate (indices of glycolysis), NEFA and glycerol were calculated 100 mg tissue^−1^ 24 h^−1^.

### mRNA abundance

mRNA abundance of *adiponectin, leptin*, *glucocorticoid receptor* (*GR*), *11β-hydroxysteroid dehydrogenase 1* (*11-βhsd1*), *adipose triglyceride lipase* (*Atgl*), *hormone sensitive lipase* (*Hsl*) and *peroxisome proliferator activated receptor γ* (*Pparγ*) relative to reference genes *tyrosine 3-monooxygenase/tryptophan 5-monooxygenase activation protein zeta* (*Ywhaz*); *Cyclin A* (*CycA*); and *ribosomal protein S9* (*S9*) was determined in explants using standard qPCR methodology (see supplementary material) and using primers in Table S2.

### Statistics

Statistical analyses were performed in RStudio (Version 0.99.893 – © 2009–2016^75^ RStudio Team, 2015). Levene’s test was used to ensure homogeneity of variance and T tests were performed to investigate sex differences in body mass, length, girth and condition. Linear mixed effects models (LME) were used to investigate effects of treatment, sex, mass and condition on biochemical and gene expression data. In addition, glucose removal rates were included as fixed effects to explain lactate, glycerol and NEFA accumulation. Relative abundance of *Atgl* and *Hsl* were included as fixed effects to explain variation in NEFA and glycerol accumulation. LMEs were fitted using maximum likelihood and included individual as a random effect to account for repeated measures. Model selection was performed using forward stepwise regression. At each iteration, the more complete model was compared with the simpler model using the ‘anova’ function and residuals plots examined. We used the ‘sem.model.fits’ function in the ‘piecewiseSEM’ package to obtain marginal and conditional R^2^ for each model^76^. The former gives the proportion of the variance explained by the fixed effects, and the latter gives the proportion of the variance explained by the fixed and random effects. The best model fit is presented in each case. To explore the relationship between indices of glycolysis and lipolysis within each treatment we performed correlations between all rates of removal and accumulation. Glycerol and lactate accumulation rates and gene expression values were log transformed for analysis.

## Electronic supplementary material

Supplementary material
