# Supplementary material for: Using blubber explants to investigate adipose function in grey seals: glycolytic, lipolytic and gene expression responses to glucose and hydrocortisone
Source: Sci Rep. 2017 Aug 10;7:7731. doi: 10.1038/s41598-017-06037-x (PMC5552887; doi:10.1038/s41598-017-06037-x)
Supplement: Supplementary file 1 — Supplementary material [file 41598_2017_6037_MOESM1_ESM.pdf]

Supplementary material: Using blubber explants to investigate adipose function in grey seals: glycolytic, lipolytic and gene expression responses to glucose and hydrocortisone

Kimberley A Bennett, Kelly J Robinson, Simon EW Moss, Sebastian Millward, Ailsa J Hall

Preparation of culture media

Explants were incubated in GIBCO™ Dulbecco's Modified Eagle Medium (DMEM) supplemented with 1% penicillin-streptomycin; 1% glutamax and 5% heat-inactivated fetal calf serum (product 10082-147), termed 'complete' media. All media contained phenol red and 1 mM pyruvate. All media, except HG, contained 5.5 mM glucose, reflecting a relatively low level of glycemia for seals [1-3], but euglycemia in humans and many other mammals. HG media was the same as C media in all respects, but contained 25 mM glucose, reflecting blood glucose measured in humans with uncontrolled diabetes and typically used to simulate extreme physiological glucose doses in tissue culture scenarios. HC media, intended to reflect typical stress-induced plasma cortisol concentrations in grey seals [4], was prepared by dissolving 18.12 mg hydrocortisone (Sigma) in molecular grade ethanol, followed by 100-fold dilution in 10 ml complete media containing 5.5 mM glucose. To produce the working solution, 0.5 ml stock were diluted in 500 ml of complete media containing 5.5 mM glucose. Media and supplements were purchased from Life technologies, Paisley, UK.

RNA extraction and cDNA synthesis

Total RNA was extracted using the QIAgen RNeasy lipid kit (QIAgen, Manchester, UK), quantified using a Nanodrop 2000 spectrophotometer (Wilmington, DE, USA), and integrity was assessed by agarose gel electrophoresis. cDNA was synthesised from 200 ng total DNA-free RNA using the QuantiTect Reverse Transcription kit (QIAgen).

Primer optimisation and cycling conditions

Primer pairs for *leptin*, *glucocorticoid receptor* (*GR*), *11β-hydroxysteroid dehydrogenase 1* (*11-βHSD1*), *adipose triglyceride lipase* (*ATGL*); *hormone sensitive lipase* (*HSL*) and *peroxisome proliferator activated receptor γ* (*PPARγ*) were designed against grey seal, Weddell seal (*Leptonychotes weddelli*) or walrus (*Odobenus rosmarus*) sequence (Table S2)

using Primer3 software [5] based on mammalian sequences available through National Center for Biotechnology Information (NCBI), focussing on highly conserved regions between the carnivores and aligned using ClustalW (https://www.ebi.ac.uk/Tools/msa/clustalw2/).We used previously designed primers [6] for *adiponectin* and three potential reference genes: *tyrosine 3-monooxygenase/tryptophan 5-monooxygenase activation protein zeta* (*YWHAZ*); *Cyclin A* (*CycA*); and *ribosomal protein S9* (*S9*). Primers were synthesised by MWG Eurofins Operon (Ebersberg, Germany) or Sigma and used at 10 µM. A Taq PCR Core kit (QIAgen) was used with each primer pair to amplify from a cDNA pool in a standard thermal cycler (Applied Biosystems® 2720, Life Technologies: 3 min at 94 °C; 35 cycles of 1 min at 94 °C, 1 min 60 °C, 1 min 72 °C; final extension of 10 min at 72 °C). PCR products were visualised on a 1.5% agarose gel in Tris-borate-EDTA (TBE) buffer to ensure a single product of the predicted size was amplified. Primer annealing temperature and performance of the selected primer pairs were tested by melt curve analysis in quantitative PCR (qPCR) using StepOne software (Applied Biosystems) under the following cycling conditions; 94 °C for 10 min; 40 cycles of 95 °C for 15 s, 60 °C annealing for 1 min; 95 °C for 15 s. Efficiency was determined for each primer pair in triplicate over a four-point log serial dilution of pooled cDNA template. Primer pairs with amplification efficiencies between 90 and 110% were selected for further use.

Selection of reference genes

Mean cycle thresholds (C_T values) from each triplicate were input into NormFinder (version 0.953)[7] and Bestkeeper [8] to identify the most suitable reference gene combination. The combination of *S9* and *CycA* was the best invariant normaliser, using both Bestkeeper (*S9*: CV =4.33%; stdev = 0.92; correlation with geometric mean = 0.968; p <0.01; *CycA*: CV = 4.72%; stdev =1.02; correlation with geometric mean = 0.973 p < 0.001; correlation between *CycA* and *S9* = 0.885, p <0.001) and Normfinder (Stability value *S9* = 0.007; *Cyc A* = 0.009; *YWHAZ* = 0.026; *S9* + *CycA* = 0.006).

qPCR

cDNA from each animal from each treatment was diluted 1:10 and used as template in triplicate reactions with iTaq Universal Master Mix (BioRad, Hertfordshire, UK) and 0.5 µl of each of 10µM forward and reverse primers. C_T values of target genes were normalised to

the geometric mean of the reference genes and normalised values were used in statistical analyses [9].

References

1. Houser, D.S., Champagne, C.D & Crocker, D.E. A non-traditional model of the metabolic syndrome: the adaptive significance of insulin resistance in fasting-adapted seals. *Front Endocrinol.* **4**, 164, 10.3389/fendo.2013.00164. (2013).

2. Bennett, K.A., Hammill, M. & Currie, S. Liver glucose-6-phosphatase proteins in suckling and weaned grey seal pups: structural similarities to other mammals and relationship to nutrition, insulin signalling and metabolite levels. *J. Comp. Physiol. B* **183**, 1075-1088. (2013).

3. Bennett, K.A., Turner, L.M., Millward, S., Moss, S.E.W. & Hall, A.J. Obtaining accurate glucose measurements from wild animals under field conditions: comparing a hand held glucometer with a standard laboratory technique in grey seals. *Conserv. Physiol*. **5**, cox013(2017).

4. Bennett, K.A., Moss, S.E.W., Pomeroy, P.P., Speakman, J.R. & Fedak, M.A. Effects of handling regime and sex on changes in cortisol, thyroid hormones and body mass in fasting grey seal pups*. Comp. Biochem. Physiol. A* **161**, 69-76. (2012).

5. Untergrasser, A., Cucutache, I., Koressaar, T., Ye, J. & Faircloth, B.C., *et al.* Primer3 – new capabilities and interfaces. *Nucleic Acids Res.* **40**, e115 10.1093/nar/gks596. (2012).

6. Bennett, K.A., Hughes, J., Stamatas, S., Brand, S., Foster, N.L., Moss, S.E.W. & Pomeroy, P.P. Adiponectin and insulin in grey seals during suckling and fasting: relationship with nutritional state and body mass during nursing in mothers and pups. *Physiol. Biochem. Zool.* **88**, 295-310. (2015).

7. Anderson, C.L., Ledet-Jensen J. & Ørntoft T. Normalization of real-time quantitative RT-PCR data: a model based variance estimation approach to identify genes suited for

normalization - applied to bladder- and colon-cancer data-sets. *Cancer Res.* **64**, 5245-5250. (2004).

8. Pfaffl, M.W., Tichopad, A., Prgomet, C. & Neuvians, T.P. Determination of stable housekeeping genes, differentially regulated target genes and sample integrity: Bestkeeper – Excel-based tool using pair-wise comparisons. *Biotech Lett* **26**, 509-515. (2004).

9. Schmittgen, T.D. & Litvak, K.J. Analysing real-time PCR data by the comparative C_T method. *Nature Protocols*. **3,** 1101-1108. (2008).

Supplementary Table S1: Rate of glucose removal, lactate, glycerol and NEFA accumulation, Ct values for reference genes (*CycA*; *S9*) and genes of interest (*Atgl; Hsl; PPARγ; 11βHSD1 (HSD); GR; adiponectin (adipo)* and *leptin*) in blubber explants from 10 adult grey seals (*Halicherous grypus*) incubated for 24 h in control (C: 5.5mM glucose), high glucose (HG: 25 mM glucose), and high hydrocortisone (HC: 5.5 mM glucose + 500 nM hydrocortisone) tissue culture media as described in the methods. – indicates where no data are available as a result of insufficient tissue or media.

			**Biochemical parameters**				**Ct values**								
**Animal**	**Sex**	**Treatment**	**Glucose (µmoles 100 mg^{-1} 24 h^{-1})**	**Lactate (µmoles 100 mg^{-1} 24 h^{-1})**	**Glycerol (nmoles 100 mg^{-1} 24 h^{-1})**	**NEFA (µmoles 100 mg^{-1} 24 h^{-1})**	***CycA***	***S9***	***Atgl***	***Hsl***	***PPARγ***	***HSD***	***GR***	***adipo***	***leptin***
1	F	C	2.63	5.64	701.96	40.00	21.54	21.81	21.65	25.28	22.53	27.56	26.39	20.62	23.34
		HG	4.87	7.06	635.23	79.83	20.00	19.88	21.78	27.20	25.53	27.72	25.76	20.52	24.45
		HC	2.97	5.83	512.50	44.81	19.48	19.23	19.57	22.53	32.04	26.51	23.51	18.37	21.58
2	M	C	3.43	6.34	794.54	89.26	21.46	20.81	22.65	27.34	25.75	32.41	26.05	21.65	24.25
		HG	4.19	9.12	876.30	108.87	21.93	21.56	22.06	26.33	25.88	29.02	26.51	21.68	24.89
		HC	3.36	6.60	803.56	93.83	23.27	22.18	24.34	29.60	26.61	32.88	27.18	23.64	25.94
3	F	C	3.65	7.75	445.42	81.6	24.49	20.46	22.40	27.38	25.99	29.58	25.99	21.39	24.65
		HG	5.24	10.65	727.16	121.32	20.38	21.05	21.10	25.84	25.08	28.29	25.02	19.91	23.87
		HC	3.19	6.24	705.98	51.23	21.30	20.81	23.04	25.99	25.90	29.57	27.89	23.57	25.72
4	F	C	2.69	5.66	554.80	63.16	19.96	19.73	19.44	22.64	23.42	26.01	22.91	17.98	22.68
		HG	4.03	8.32	719.89	48.48	19.64	19.65	19.91	24.56	24.70	26.20	23.39	17.97	23.44
		HC	3.15	5.00	610.33	24.19	19.61	20.24	18.34	21.85	22.51	24.94	22.69	16.81	20.57
5	F	C	2.84	6.47	516.13	90.00	20.84	20.37	21.69	25.39	25.50	28.33	24.30	20.45	23.36
		HG	5.08	9.18	578.18	109.77	21.71	21.21	21.05	25.04	24.98	28.56	24.24	20.43	23.17
		HC	2.58	5.53	504.64	64.73	21.66	20.47	22.05	26.12	25.23	28.74	25.20	21.33	23.82
6	F	C	1.78	4.40	432.30	93.76	21.06	21.40	21.76	25.43	24.94	28.46	24.76	21.48	25.33
		HG	2.24	5.81	645.45	108.79	21.85	21.84	21.86	27.55	28.65	28.66	24.15	21.81	26.36
		HC	2.56	5.15	458.52	64.37	21.28	20.39	21.37	25.41	24.36	28.47	24.38	21.59	25.99
7	M	C	4.41	9.42	770.14	128.39	22.54	22.88	23.38	27.53	26.29	32.14	26.70	24.38	26.63
		HG	5.55	11.96	983.03	102.49	21.88	21.79	20.41	24.58	24.09	28.91	25.10	19.06	24.42
		HC	1.36	5.04	579.70	102.35	22.04	21.66	23.96	27.77	26.77	30.62	26.10	22.82	25.67
8	M	C	-	-	-	-	20.99	21.05	23.02	27.17	26.00	28.73	25.03	21.61	25.21
		HG	6.31	13.39	1291.97	47.21	19.94	20.39	20.67	24.75	24.40	27.47	24.69	19.31	22.93
		HC	3.69	6.70	593.33	80.16	21.03	21.24	21.08	24.18	25.03	29.89	26.65	20.96	23.67
9	M	C	-	-	-	-	21.91	21.84	23.4	26.98	27.26	31.36	27.29	-	-
		HG	3.93	8.90	924.32	96.19	21.35	21.65	21.68	25.53	26.93	29.14	26.39	-	-
		HC	1.46	4.16	434.89	76.8	22.11	-	22.67	26.21	25.99	-	-	-	-
10	M	C	-	-	-	-	21.29	21.39	21.27	23.95	27.13	29.93	26.34	-	-
		HG	4.69	8.99	712.67	131.35	20.62	20.62	20.19	20.93	24.32	27.57	25.71	-	-

HC	2.29	5.25	516.72	165.07	22.30	22.3	22.05	24.75	27.01	30.26	26.84	-	-

Supplementary Table S2: 5′ to 3′ sequences of forward and reverse primers used to amplify each reference and target gene from grey seal blubber, their size and efficiency of the qPCR reaction. R^2 in each case was > 0.99. Primers validated and used previously [6] are indicated.

Gene	Forward	size (bp)	Efficiency (%)
Atgl	F: ACGCTATGTTGATGGTGGCA<br>R: ATGGATGTTGCTGGAGCTGTC	83	96.7
Hsl	F: ACCTCCAAATCCCATGAGCC<br>R: GTAGAAGCACTCCTCCAGCG	87	97.98
Pparγ	F: TCCAGCATTTCCACTCCACA<br>R: GCAGGCTCCACTTGGATTGC	82	108.36
11-βHSD1	F: TGGCCTATCATCTGGCGAAA<br>R: TGGAGCCAGCAATGTAGTGT	90	90
Gr	F: AGGAATTCAGCAGGCCACTC<br>R: TGGTCATGATCCGCCAAGTC	134	90
Adiponectin[6]	F: TATGATGGCACCACTGGAAA<br>R: GCCTGGTCCACATTCTTCTC	164	93.38
Leptin	F: CCCATCCAAAGAGTTCAGGA<br>R: TAGACCAGCAACCCTTGGTC	74	95.5
Ywhaz[6]	F: GAGGTTGCTGCTGGTGATGA<br>R: TCCGGGGAGTTCAGAATTTCG	170	91.61
CycA[6]	F: TCATCTGCACCGCCAAGAC<br>R: AAGCGCTCCATGGCTTCCAC	260	93.2
S9[6]	F: ACATCCCGTCCTTCATTGTC<br>R: CAATCCTCCTCCTCGTCATC	157	101.44
